# Diseases of the Liver

**Published:** 1895-01-05

**Authors:** 


### PROGRESS IN SURGERY.

### DISEASES OF THE LIVER.

**Tropical Abscess of the Liver.**—Dr. Neil Macleod[1] raises the old question of the association of dysentery and hepatic abscess. He says that in every fatal case of hepatic abscess examined post-mortem, he has found dysenteric ulcers and their cicatrices. He also quotes a case showing, that although numerous ulcers may be present, there need not be the characteristic diarrhœa of dysentery. Dr. Macleod's communication led to some correspondence. Surgeon-Lieutenant Yeates, of Mandalay,[2] denied the influence of dysentery, stating that although that disease is common in the natives of Burmah, hepatic abscess rarely occurs. Hepatic abscess, on the other hand, is not uncommon in Europeans, who render their livers susceptible to inflammation by eating and drinking as they did before they left their native countries, instead of adapting their mode of life to their new surroundings. Surgeon-Major Younge also disagreed with the opinion of Dr. Macleod, saying that post-mortem examination often showed that no dysentery had been present.[3] Surgeon-General Jessop and Surgeon-Major Rennie also contributed to the discussion, and Dr. Macleod replied, but no facts of great importance were mentioned. The scientific work of Kruse, Pasquale, and others, leads to somewhat more definite conclusions than ordinary post-mortem investigation. The above-mentioned writers[4] carefully studied fifteen cases of abscess of the liver, and in six a distinct connection with dysentery could be made out. In these six abscesses the amœba, that is now considered to be the cause of dysentery, was found. In connection with this subject, a case reported by Dr. Patrick Manson[5] is of some interest, since so far as Dr. Manson is aware, it is the first case in England in which the amœba of dysentery has been found in pus from a liver abscess. The patient was a sailor, a native of Zanzibar, employed on steamers running from Bombay to London. The abscess was opened, and Dr. Manson found living amœbæ in the pus in abundance.

Dr. Hale White describes[6] a case of abscess of the liver associated with ulcerative colitis in a man aged 43, who had never been out of England.

Clinically, the case was thought possibly one of suppurating hydrated cyst. At the time of the operation by Mr. Lane, however, it was found to be an abscess of the liver. The pus was repeatedly searched for amœbæ, but none were found. Since, however, the man had not been out of England, their presence was not expected. The man gradually sank and died, and at the post-mortem examination, in addition to the large abscess opened during life, the presence of other smaller abscesses was discovered in the liver. The cæcum and the ascending colon were ulcerated. Dr. Hale White discusses the connection between the abscess and the ulcerated colitis, mentioning that although tropical dysentery and abscess of the liver are frequently associated, the English form of ulceration of the colon is rarely complicated by liver abscess. Dysentery and the associated liver abscess both owe their existence to the same cause, the amœba coli, but the cause of ulcerative colitis is unknown, and a liver abscess, where present, is probably not due to the cause of the colitis, whatever that may be, but to septic infection from the denuded surface of the mucous membrane.

**Cirrhosis of the Liver.**—Some French writers believe that alcohol is not the only poison that will set up cirrhosis of the liver, and Hanot and Boix describe a form of moderate hypertrophy associated with prolonged dyspepsia,[7] which they attribute to the absorption of toxines. Alcoholism, they say, can be definitely excluded, while tuberculosis, syphilis, malaria, and other infectious diseases cannot be demonstrated. There is, however, a history of digestive derangement lasting several years sometimes associated with gastric dilatation. The liver extends in advanced cases several fingers' breadth below the costal margin, presents a smooth hard surface, and a thickened well-defined margin. Palpation causes no pain. The spleen is not enlarged and there is no ascites. After attaining a certain size the liver ceases to enlarge and apparently causes little inconvenience. Lassitude and a sense of weight in the right hypochondrium are the most obvious symptoms. Histological examination shows the sclerosis to be in the interlobular connective tissue.

**Hepatic Cirrhosis with Enlarged Spleen.**—Dr. E. Banti, of Florence, describes a form of cirrhosis associated with enlargement of the spleen.[8] The disease, which progresses in an insidious manner appears to commence with hypertrophy of the spleen. Anæmia and hæmic cardiac murmurs are present, but there is no leucocytosis and no deformity nor abnormal size of the red blood corpuscles. At first there are no gastro-intestinal disorders, but these supervene as the disease progresses. Hepatic cirrhosis and ascites occur before death ends the case. Patients affected with this disease have been endowed with a good constitution, and have not suffered from syphilis, malaria, or other infectious diseases. Dr. Banti thinks that the disease probably originates in the spleen, and that, either through some abnormal bio-chemical process or in the presence of an infective agent, toxic substances are produced that enter the blood, cause anæmia, and later hepatic cirrhosis.

**Cirrhosis in Children.**—Dr. Michell Clarke[9] describes two cases of cirrhosis in children, in which alcohol and syphilis as causes could be excluded. There was also, on post-mortem examination, no evidence of tubercle. Dr. Clarke quotes Henoch as stating that in a few cases of cirrhosis in children the cause remains unknown. Dr. Clarke suggests that in his two cases the absorption of some toxine from the intestinal canal set up the interstitial inflammation.

It may not be out of place here to refer to the antitoxic action of the liver. Schupfer, of Rome,[10] has investigated the power of the liver to destroy poisonous substances by experiments upon the frog. The frog will live some days after the removal of the liver, and various alkaloids were injected into healthy animals, and into others that had been deprived of their livers. The result of these experiments has led Dr. Schupfer to conclude that the liver has the power of diminishing the toxicity of poisons introduced into the system, and also of others manufactured in the body in consequence of putrefactions or the activity of tissues. Professor Bouchard[11] refers to the work of M. Pavlow upon the same subject. M. Pavlow found that when the blood of the portal circulation was forced by ligature of the portal vein to enter the general circulation poisonous symptoms appeared.

A communication by Dr. Hall Calvert[12] upon an epidemic of jaundice, which he considered to have been associated with influenza, led to considerable correspondence, Dr. Pope Bartlett, Dr. Semple Young, and Dr. Cullen sending letters, and Dr. Rankin a paper upon the subject. Dr. Calvert afterwards quoted a paper by Meinert, of Dresden, in which 180 cases of jaundice were mentioned, the majority occurring after influenza. Dr. Semple Young considered his cases to have been connected with influenza, but Dr. Pope Bartlett and Dr. Rankin could establish no connection with that disease, and Dr Cullen argued strongly against such an explanation of three epidemics that occurred in his district. Jaundice in the epidemic form attacks children most frequently, adults comparatively rarely. All the younger members of one family may suffer. The disease is of simple nature, and does not appear to be sufficiently severe to necessitate confinement to bed.

**The Liver and the Formation of Urea.** — Professor Kaufmann[13] has estimated the amount of urea in the blood and various tissues of the body, and has found that the liver contains the greatest proportion of this substance. From his experiments he concludes that the formation of urea is not limited to the liver, but that that glandular organ is the main seat of its production. Professor Richet[14] has also found the quantity of urea in the liver to be large, and says that it increases for some hours after the death of the individual. He refers to the formation of sugar from glycogen, and considers that both sugar and urea are the products of the action of a soluble diastase. Münzer[15] criticises the generally accepted view as to the liver being the seat of the formation of urea. He does not deny that in birds removal of the liver causes the formation of uric acid to cease, but he states that in the dog-fish extirpation of the liver has no influence upon the amount of urea in the muscles, and that if ammonium salts are injected into the lymph sacs of frogs, in which the livers have been removed, urea appears in the excrement. Münzer does not deny the urea-forming power of the liver, but thinks that it is probable that every organ possesses urea-forming function, according to the activity of its metabolism.

**The Treatment of Hepatic Colic.**—Dugardin - Beaumetz[16] refers to the numerous papers upon the value of olive oil in gall-stone colic, and mentions that although there appears to be little doubt about the success of the treatment, the large quantities of oil are repugnant to the patients. He considers glycerine in doses of two and a half to five drachms to be equally effective. Terrand[17] also speaks of the value of glycerine, but recommends from half to one ounce during an attack of colic.

[1] B.M.J., Mar. 31. [2] B.M.J., Aug. 15. [3] B.M.J., Aug. 25. [4] Am. J. Med. Sc., Sept. [5] B.M.J., Mar. 31. [6] Lanc., Mar. 31. [7] Wiener Med. Presse, No. 15, p. 577. [8] Med. Week. Aug. 3. [9] B.M J., Jan. 30. [10] Lanc., Mar. 17 [11] Glasgow Med. J., May. [12] B.M.J., Feb. 3. [13] Med. April 27. [14] Med. Week, May 4 and June 1. [15] N.Y. Med. J., Aug. 25. [16] Am. J. Med. Sc., Sept. [17] L'Union Med., May 8.

